# Misplaced Ovary

**Published:** 1868-04

**Authors:** Edward Whinery

**Affiliations:** Ft. Madison, Iowa


### MISPLACED OVARY.

BY EDWARD WHINERY, M. D., FT. MADISON, IOWA.

Read before the Iowa State Medical Society, at its session in Davenport, May, 1867.

IN May, 1861, a maiden lady of 26 years of age, (Miss L.) consulted me in regard to what appeared to be a cist or wen in the right *labium major.* She said that when first noticed by her it was about the size of a large bean, but that about six years ago it began to increase in size, and that recently it had been growing more rapidly, so that it had attained apparently the size of a walnut, and it somewhat interfered with walking. There was no appearance of diseased action. It was firm to the touch, but could be moved backward toward the posterior commissure.

I advised extirpation as the proper and safe thing to be done. She left my office without saying whether she would have the operation performed, but in a few days I was asked to see her at Dr. Wm. Reinhart's, whom she had first consulted, but did not tell me of it. I now learned that our diagnosis, without consultation, agreed, and that she had come to town to be operated on.

I made a sweep of the scalpel in the line of the labium over testicle. It had a beautiful tunic, resembling the *tunica albuginia* of a testicle, and a tunic corresponding to the *tunica vaginalis testis.* I said to Dr. R., we must proceed carefully; there will be a pedicle to ligate. A ligature was procured, and I enlarged the incision in the *tunica vaginalis*, and the misplaced *ovary* turned out beautifully. It was of a kidney shape, with a pedicle entering at its pelvis. This was ligated and then divided. Its length was two inches, its width an inch and a half, and its thickness one inch. The wound healed mostly by the first intention. The ligature came away in six days, but there remained a small fistulous opening that discharged occasionally a semi-purulent matter for about six weeks. It is uncertain at what period in life it made its descent through the inguinal canal, but it is probable that it was in early infancy.

She told me a year after the operation, that she only menstruated half as much as formerly. She is a fine healthy woman, yet unmarried.

